# Genomic classification and outcomes of young patients with polycythemia vera and essential thrombocythemia according to the presence of splanchnic vein thrombosis and its chronology

**DOI:** 10.1007/s00277-023-05610-x

**Published:** 2024-01-24

**Authors:** Marta Garrote, Mónica López-Guerra, Juan Carlos García-Pagán, Eduardo Arellano-Rodrigo, Francisca Ferrer-Marín, Juan Carlos Hernández-Boluda, Beatriz Bellosillo, Meritxell Nomdedeu, Virginia Hernández-Gea, Ana Triguero, Francesca Guijarro, José Álamo, Anna Baiges, Fanny Turon, Dolors Colomer, Francisco Cervantes, Alberto Alvarez-Larrán

**Affiliations:** 1 Facultat de Medicina, Universitat de Barcelona, Barcelona, Spain; 2 Hematopathology Unit, Pathology Department, Hospital Clínic, Barcelona, Spain; 3 Institut d’Investigacions Biomèdiques August Pi i Sunyer (IDIBAPS), Barcelona, Spain; 4 Centro Investigación Biomédica en Red (CIBER) Oncología (CIBERONC) y Enfermedades Hepáticas y Digestivas (CIBEREHED), Madrid, Spain; 5 Barcelona Hepatic Hemodynamic Lab, Liver Unit, Hospital Clínic, Barcelona, Spain; 6 Hemotherapy and Hemostasis Department, Hospital Clínic, Barcelona, Spain; 7 Hematology Department, Hospital Morales-Messeguer, Murcia, Spain; 8 Hematology Department, Hospital Clínico-INCLIVA, Valencia, Spain; 9 Pathology Department, Hospital del Mar, Barcelona, Spain; 10 Hematology Department, Hospital Clínic, Barcelona, Spain

## Abstract

To elucidate the role of splanchnic vein thrombosis (SVT) and genomic characteristics in prognosis and survival, we compared patients with polycythemia vera (PV) or essential thrombocythemia (ET) presenting SVT at diagnosis (*n* = 69, median age 43 years) or during follow-up (*n* = 21, median age 46 years) to a sex- and age-matched control group of PV/ET without SVT (*n* = 165, median age 48 years). The majority of patients presenting with SVT at diagnosis were classified as myeloproliferative neoplasm with heterozygous *JAK2* mutation (87% of cases vs. 69% in PV/ET control group, *p* < 0.05), characterized by low *JAK2* allele burden and no high-risk mutations. Despite this lower molecular complexity, patients presenting with SVT showed a higher risk of death (HR 3.0, 95% CI 1.5-6.0, *p* = 0.003) and lower event-free survival (HR 3.0, 95% CI 1.9–4.8, *p* < 0.001) than age- and sex-matched PV/ET controls. In patients presenting with SVT, molecular high-risk was associated with increased risk of venous re-thrombosis (HR 5.8, 95% CI 1.4–24.0, *p* = 0.01). Patients developing SVT during follow-up were more frequently allocated in molecular high-risk than those with SVT at diagnosis (52% versus 13%, *p* < 0.05). In the whole cohort of patients, molecular classification identified PV/ET patients at higher risk of disease progression whereas *DNMT3A/TET2/ASXL1* mutations were associated with higher risk of arterial thrombosis. In conclusion, clinical and molecular characteristics are different in PV/ET patients with SVT, depending on whether it occurs at diagnosis or at follow-up. Molecular characterization by NGS is useful for assessing the risk of thrombosis and disease progression in young patients with PV/ET.

**Supplementary Information:**

The online version contains supplementary material available at 10.1007/s00277-023-05610-x.

## Introduction

Individuals with myeloproliferative neoplasms (MPN), including polycythemia vera (PV), essential thrombocythemia (ET) and primary myelofibrosis (MF), are at increased risk of thrombosis, which can involve unusual sites such as the splanchnic area [1–3]. Approximately 5% of MPN have splanchnic vein thrombosis (SVT) at presentation or during follow-up and it has been postulated that these patients have specific demographic and phenotypic features including younger age, female predominance and a milder hematological picture, with a complete blood count closer to normal values [4–6].

It has been suggested that MPN with SVT have a differentiated genomic background: more than 95% of MPN presenting with SVT harbor *JAK2* V617F regardless of MPN subtype. In addition, a lower allele frequency (VAF) of the driver mutation is described, if compared with MPN patients without SVT. However, whether these findings are associated with lower molecular complexity is not fully understood since studies including a comparison with age and sex-matched MPN patients and taking SVT chronology into account are lacking [5–7]. Moreover, contradictory results have been reported in relation to survival and disease progression of MPN patients with SVT due to design heterogeneity of published works, regarding type of underlying MPN, SVT chronology or control groups employed for comparison [4–6, 8, 9].

The aim of the present study is to assess clinical and molecular characteristics of a cohort of PV/ET with SVT taking into consideration chronology of SVT. For such purpose, main clinical outcomes and molecular data derived from targeted Next-Generation Sequencing (NGS) are compared with a sex- and age-matched PV/ET control group without SVT.

## Methods

### Patient selection and data collection

Ninety-seven patients with SVT were selected from a database of patients diagnosed with MPN in our institution (from 1995 to 2022). SVT patients with primary myelofibrosis (*n* = 3), MPN without *JAK2* mutation (*n* = 3) and patients with other diagnosis (*n* = 1) were excluded due to heterogeneity and low representation. The final number of MPN with SVT was 90. Selected cases did not have other factors that may explain predisposition to SVT before MPN diagnosis, such as liver cirrhosis, cancer, known thrombophilia or paroxysmal nocturnal hemoglobinuria. In relation to SVT chronology, in MPN with previous SVT, SVT developed at least 6 months before MPN diagnosis; and in MPN with SVT during follow-up, SVT occurred at least 6 months after MPN diagnosis.

A matched control group was constituted, including 1 or 2 *JAK2*-mutated MPN patients without SVT for every SVT case, with same sex, similar age at diagnosis (a maximum of a 5-year difference was allowed) and same MPN diagnosis (PV or ET). Cases presenting with SVT and diagnosed with MPN, unclassifiable (MPN-U), were matched with one PV case and one ET case. MPN-U cases without SVT are notably uncommon and typically exhibit distinct characteristics; hence, they were considered unsuitable for the matching. The final control group comprised 165 MPN patients without SVT. MPN diagnosis was established based on World Health Organization criteria (2016). Main clinical data at MPN diagnosis, including age, sex, cardiovascular risk factors and complete blood count, besides complications during evolution (thrombosis, bleeding and progression to myelofibrosis or acute leukemia) and status at last follow-up were collected. Signed informed consent according to local institutional review board requirements was obtained from all participants.

### Molecular analysis

DNA from peripheral blood at diagnosis (*n* = 192, 75%) or during follow-up (*n* = 63, 25%) and stored in Biobank Hospital Clínic (R121004-094) was used. Targeted sequencing using a 32-gene customized commercial panel from Sophia Genetics was performed using Illumina MiSeq platform (see Supplemental Table S1). GRCh37 (hg19) was used as reference genome. Variant calling was performed using *SOPHiA DDM™*. Only variants with a VAF ≥ 1% were considered and classified based on information from databases (COSMIC, ClinVar, Seshat, Franklin and Varsome) and published works on myeloid neoplasms. Only pathogenic/likely pathogenic variants were taken into account for further analysis.

Subsequently, genomic classification proposed by Grinfeld et al. [10] – referred from this point as the Grinfeld algorithm – was applied to every case, according to mutational data. Briefly, patients were hierarchically allocated into four molecular subgroups: *TP53* disruption or aneuploidy (*TP53* mutation, Chr17pLOH or Chr5-/Chr5q-); ≥1 genetic aberrations in chromatin or spliceosome genes (*EZH2*, *IDH1*, *IDH2*, *ASXL1*, *PHF6*, *CUX1*, *ZRSR2*, *SRSF2*, *U2AF1*, *KRAS*, *NRAS*, *GNAS*, *CBL*, Chr7/7qLOH, Chr4qLOH, *RUNX1*, *STAG2* and *BCOR*); homozygous *JAK2* mutation (allele frequency ≥ 50% or Chr9pLOH, when available); and heterozygous *JAK2* mutation. Patients belonging to heterozygous *JAK2* mutation group were classified as molecular low-risk whereas patients allocated in the other three groups were considered as molecular high-risk. Pathogenic or likely pathogenic mutations in *DNMT3A*, *TET2* or *ASXL1* genes were defined as DTA mutations.

### Statistical analysis

Qualitative variables were compared through Chi-squared test and quantitative variables through Student’s t-test, Mann-Whitney U test or ANOVA. Overall survival and time-to-event curves were drawn using the method of Kaplan-Meier with log-rank test for comparisons. Event-free survival was calculated as time free of thrombosis, major bleeding, disease progression or death, whichever occurred first. Variables evaluated for their potential prognostic significance were age, sex, history of SVT, hematological values at diagnosis, genomic classification and DTA mutations. Multivariate analyses of the factors predicting the different outcomes were done by Cox regression. All statistical analyses were carried out with R and SPSS software.

## Results

### General characteristics

A total of 255 patients were included (PV, *n* = 159; ET, *n* = 75; MPN-U, *n* = 21). Ninety patients developed SVT: 59 at MPN presentation, 10 before MPN diagnosis and 21 during follow-up. The remaining 165 patients were used as controls. Regarding SVT localization, in 58 patients (64%) affected the splenoportal area, 29 patients (32%) had Budd-Chiari syndrome, and in 3 patients (3%) comprehended both territories.

The main clinical and hematological features at diagnosis, according to the presence of SVT and its chronology, are shown in Table 1. There were no significant differences among cases and controls regarding age (median age at MPN diagnosis, 47 y-o) and sex (female sex, 56%), as groups were balanced on purpose. A higher proportion of patients with MPN-U were observed among MPN presenting with SVT. In such cases, a normal blood count was the reason to establish MPN-U diagnosis whereas in controls the small proportion of patients with MPN-U had phenotypic characteristics between PV and ET.

Patients presenting with SVT showed lower hemoglobin level and platelet count and higher frequency of splenomegaly. Those with evolutive SVT showed similar clinical characteristics at diagnosis than PV/ET controls, except for higher frequency of splenomegaly (*p* < 0.0001) (Table 1).

**Table 1:** Main clinical and hematological characteristics at MPN diagnosis in 90 MPN with SVT and 165 age- and sex-matched MPN controls

	MPN without SVT, *n* = 165	MPN presenting with SVT or previous SVT¶, *n* = 69	MPN with evolutive SVT, *n* = 21	*p* value†
Age*	48 (37–60)	43 (36–56)	46 (33–55)	0.47
Female sex, *n* (%)	91 (55)	41 (59)	10 (48)	0.61
Type of MPN				0.003
PV, *n* (%)	106 (64)	41 (59)	12 (57)	
ET, *n* (%)	53 (32)	15 (22)	7 (33)	
MPN-U, *n* (%)	6 (4)^2^	13 (19)^1^	2 (9.5)	
CVRF	49%	48.5%	57%	0.7
Hemoglobin level, g/L*	161 (150–180)^2^	143 (123–164)^1^	157 (143–172)	< 0.001
Leukocyte count, ×10^9^/L*	9.4 (7.8–11.7)	8.3 (6.6–12)	11.1 (8.6–13.5)	0.26
Platelet count ×10^9^/L*	660 (463–760)^2^	414 (245–499)^1^	482 (303–662)	< 0.001
Pruritus	17%	7%^3^	36%^2^	0.02
Microvascular symptoms	26%^2^	5%^1^	7%	0.002
Palpable spleen	13%^2,3^	42%^1^	40%^1^	< 0.001
Previous/at diagnosis hemorrhage	4.2%	19%	4.8%	< 0.001
Previous/at diagnosis non-SVT thrombosis	13%	13%	29%	0.14
High-risk according to the ELN‡, n (%)	38%	100%	48%	< 0.001

*Abbreviations*: MPN, myeloproliferative neoplasm; SVT, splanchnic vein thrombosis; PV, polycythemia vera; ET, essential thrombocythemia; MPN-U, myeloproliferative neoplasm, unclassifiable; CVRF, cardiovascular risk factors; ELN, European LeukemiaNet

*Median (Interquartile range); ‡High-risk defined as age ≥ 60 years-old and/or history of thrombosis or hemorrhage

¶Includes 59 patients presenting with SVT and 10 patients with history of SVT prior to MPN diagnosis

†*p* value, as a result of comparison of the three groups

^1^*p*<0.05 in comparison with MPN without SVT

^2^*p*<0.05 in comparison with MPN presenting with SVT

^3^*p*<0.05 in comparison with MPN with evolutive SVT

### Molecular features and classification

**Fig. 1:**
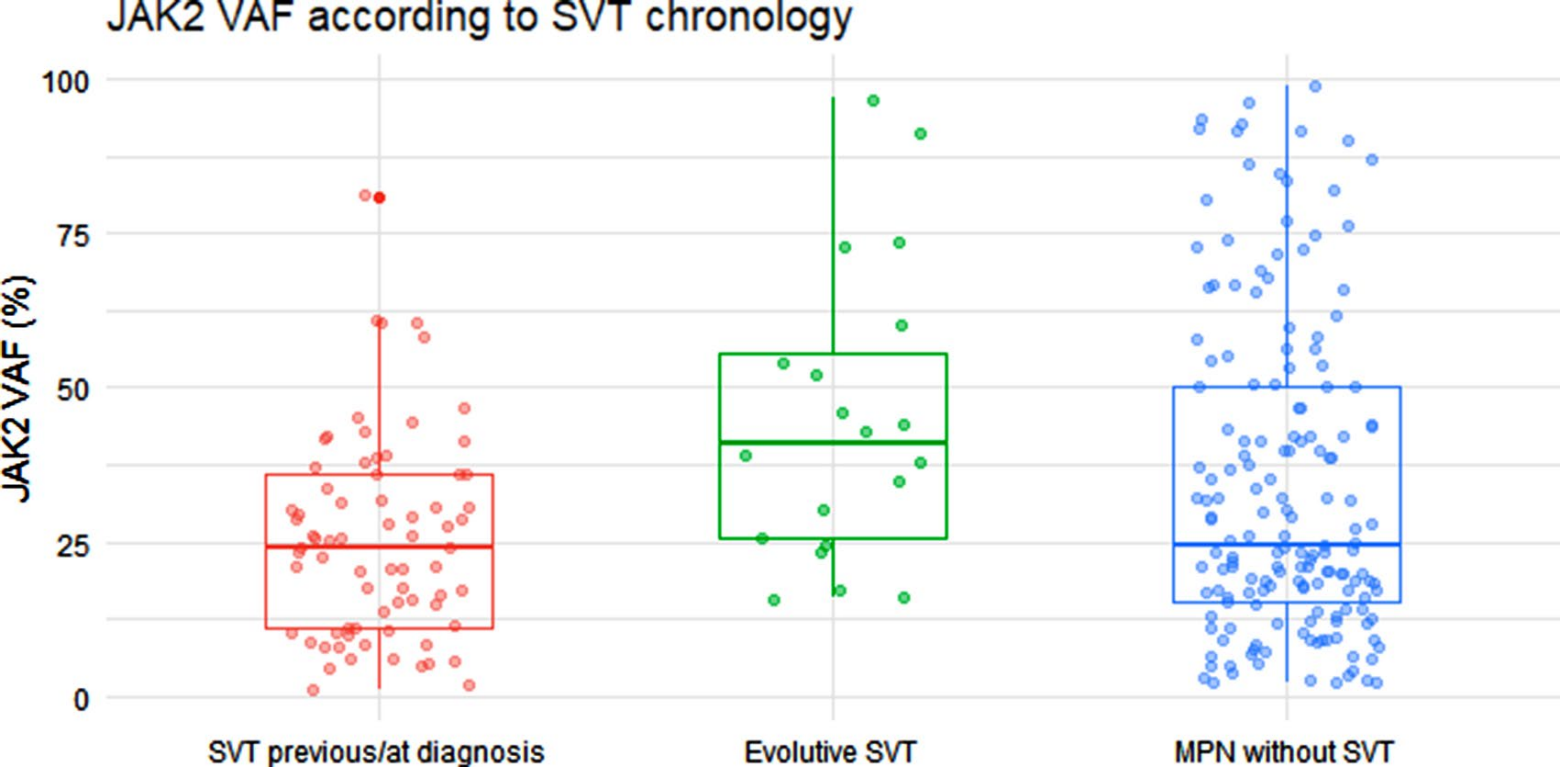
*JAK2* VAF (%) in 255 MPN patients separated according to SVT chronology. This box plot plus jitter shows *JAK2* VAF distribution according to SVT chronology. Only very few cases of SVT at MPN diagnosis or previously presented a VAF ≥ 50%, which is very different from MPN without SVT. MPN with evolutive SVT showed the highest *JAK2* allele VAF. *Abbreviation*: VAF, variant allele frequency; MPN, myeloproliferative neoplasm; SVT, splanchnic vein thrombosis

The main molecular characteristics of the patients according to the presence of SVT and its chronology are shown in Table 2. *JAK2* V617F VAF was significantly different between groups (Fig. 1; Table 2). Patients presenting with SVT showed significant lower *JAK2* VAF than age- and sex-matched patients without SVT. Patients developing SVT during follow-up showed the highest *JAK2* VAF, with the differences being statistically significant in comparison with patients presenting with SVT but not with those without SVT (Fig. 1; Table 2).

**Table 2:** Main molecular characteristics of 90 MPN patients with SVT and 165 age- and sex-matched MPN controls

	MPN without SVT, *n* = 165	MPN presenting with SVT or previous SVT,* n* = 69	MPN with evolutive SVT, *n* = 21	*p* value†
*JAK2* VAF*	34 (30–38)^2^	25 (21–29)^1,3^	45 (34–56)^2^	0.001
Number of mutations*	1.3 (1.2–1.4)^3^	1.2 (1.1–1.3)^3^	1.7 (1.1–2.1)^1,2^	0.03
Additional mutation, *n* (%)	42 (25.5)	13 (19)	6 (29)	0.5
*TP53* mutation, *n* (%)	7 (4)^3^	2 (3)^3^	4 (19)^1,2^	0.009
Chromatin/spliceosome mutation, *n* (%)	11 (7)	2 (3)^3^	4 (19)^2^	0.03
Homozygous *JAK2* or mutation in *TP53*/chromatin/ spliceosome genes,* n* (%)	51 (31)^2^	9 (13)^1,3^	11 (52)^2^	0.001
DTA mutation, *n* (%)	34 (21)	9 (13)	4(19)	0.6
*TET2*, *n* (%)	21 (13)	6 (9)	2 (9.5)	0.8
*DNMT3A*, *n* (%)	6 (4)	2 (3)	2(9.5)	0.5
*ASXL1*, *n* (%)	11 (7)	1 (1)	0 (0)	0.2

*Abbreviation*: MPN, myeloproliferative neoplasm; SVT, splanchnic vein thrombosis; VAF, variant allele frequency; DTA, *DNMT3A, TET2, ASXL1*

*Mean (95%CI). †*p* value, as a result of comparison of the three groups

^1^*p*<0.05 in comparison with MPN without SVT

^2^*p*<0.05 in comparison with MPN presenting with SVT

^3^*p*<0.05 in comparison with MPN with evolutive SVT

There were no significant differences in the number of pathogenic/likely pathogenic variants among patients presenting with SVT and age- and sex-matched MPN without SVT. Patients developing SVT after diagnosis showed a significantly higher number of pathogenic/likely pathogenic mutations than patients presenting with SVT (Table 2). *TP53* mutations and spliceosome/chromatin mutations were significantly more frequently observed in the group of patients developing SVT during follow-up (Table 2). There were no significant differences among groups regarding the frequency of DTA mutations (Table 2).

**Fig. 2:**
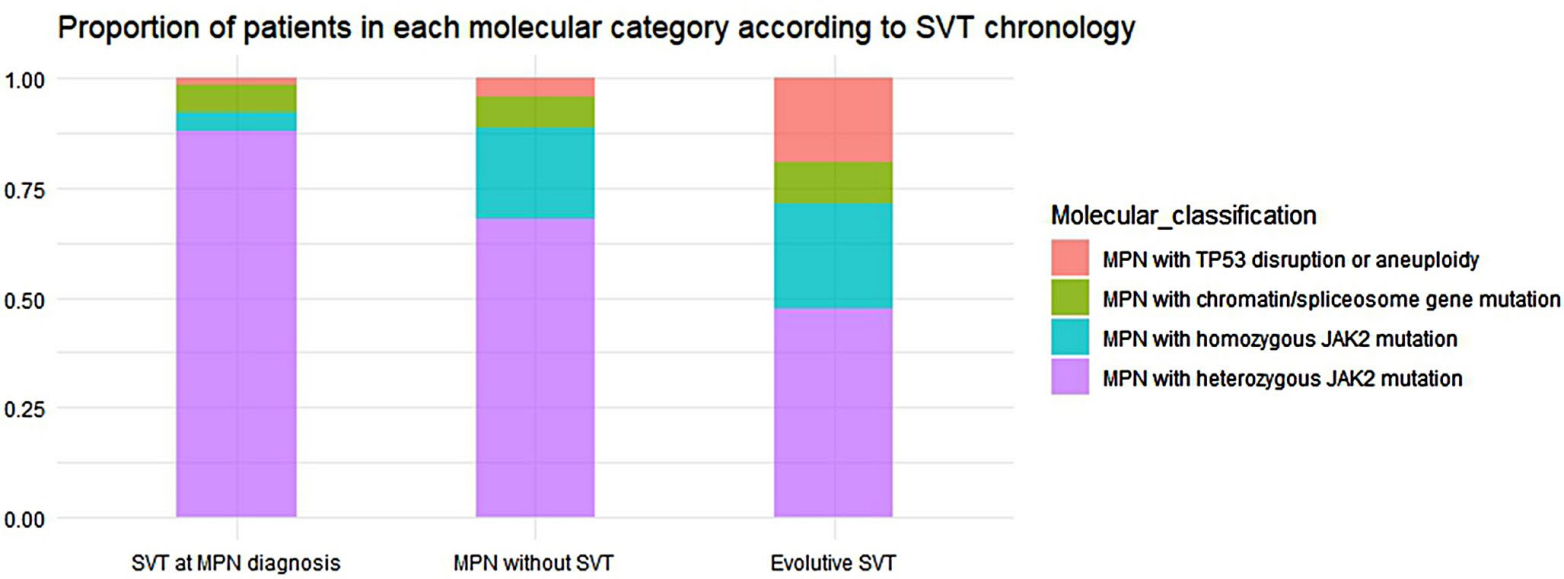
Proportion of patients in each molecular category according to SVT chronology. The bar plot shows the difference on the proportion of patients in each molecular category according to SVT chronology. MPN presenting with SVT group showed a low proportion of cases in high-risk categories (1.7% *TP53* disruption or aneuploidy, 3.4% chromatin/spliceosome gene mutation and 5.1% homozygous *JAK2* mutation) and a very high proportion of patients falling into heterozygous *JAK2* mutation category. The proportions differed from those in MPN without SVT (4.2% *TP53* disruption or aneuploidy, 6.1% chromatin/spliceosome gene mutation, 20.6% homozygous *JAK2* mutation and 69.1% homozygous *JAK2* mutation) and those in MPN with evolutive SVT (19% *TP53* disruption or aneuploidy, 9.5% chromatin/spliceosome gene mutation, 23.8% homozygous *JAK2* mutation and 47.6% heterozygous *JAK2* mutation). The differences seen on the proportion of patients in each category were statistically significant (*p* = 0.006). *Abbreviation*: MPN, Myeloproliferative neoplasm; SVT, Splanchnic vein thrombosis

The stated divergences in *JAK2* allele burden and the presence of *TP53*, spliceosome or chromatin gene mutations resulted in significant differences in relation to molecular classification according to Grinfeld algorithm (Fig. 2). Of note, 87% of cases presenting with SVT fell into MPN with heterozygous *JAK2* mutation category indicating less molecular complexity and this was significantly different from patients without SVT. Cases developing SVT during follow-up was the group with the highest molecular complexity with only 48% of them being classified as MPN with heterozygous *JAK2* mutation.

### Survival analysis

With a median follow-up of 10 years, and without significant differences regarding follow-up time between groups, 39 patients died. Causes of death according to the presence or absence of SVT and its chronology are shown in Table 3.

**Table 3:** Causes of death in 90 MPN patients with SVT and 165 age- and sex-matched MPN controls

	MPN without SVT, *n* = 165	MPN presenting with SVT, *n* = 69	MPN with evolutive SVT, *n* = 21
Thrombosis	0	0	2 (9.5)
Bleeding	1 (0.6)	3 (4.3)	1 (4.8)
Hepatic disease	0	3 (4.3)	0
Infection	2 (1.2)	3 (4.3)	1 (4.8)
MF/AML	5 (3)	1 (1.4)	1 (4.8)
Neoplasia	4 (2.4)	5 (7.2)	0
Other/Unknown	6 (3.6)	1 (1.4)	0
Total	18 (11)	16 (23)	5 (24)

*Abbreviation*: MPN, myeloproliferative neoplasm; SVT, splanchnic vein thrombosis; MF, myelofibrosis; AML, acute myeloid leukemia

**Fig. 3:**
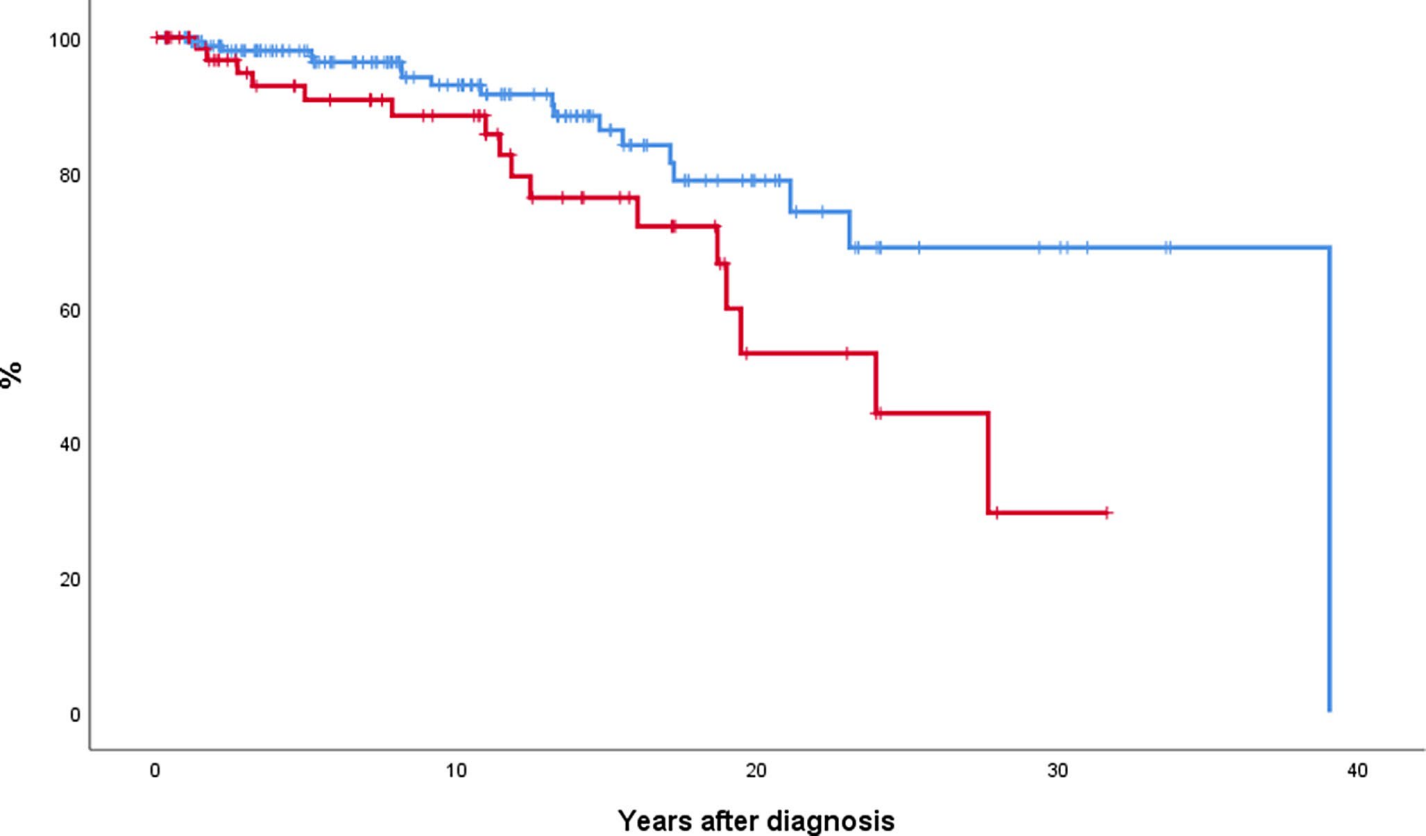
Probability of survival in 69 MPN patients presenting with SVT (red line) and 165 age- and sex-matched patients without SVT at diagnosis (blue line). Median survival was 24 and 39 years for SVT and non-SVT groups, respectively (*p* = 0.02). *Abbreviation*: MPN, myeloproliferative neoplasm; SVT, splanchnic vein thrombosis

Median survival was 24 and 39 years in patients with SVT and MPN without SVT, respectively (*p* = 0.038). Results were similar when patients developing SVT during follow-up were excluded from the analysis (Fig. 3). Molecular high-risk patients also showed a shorter survival than patients with heterozygous *JAK2* mutation (median survival 23 years and not reached, respectively, *p* = 0.036). At multivariate analysis, patients presenting with SVT showed a significant higher risk of death (HR 3.0, 95% CI 1.5-6.0, *p* = 0.003) after correction by age (HR 1.1, 95% CI 1.08–1.15, *p* < 0.001), sex (not significant), and molecular risk (not significant)

**Fig. 4:**
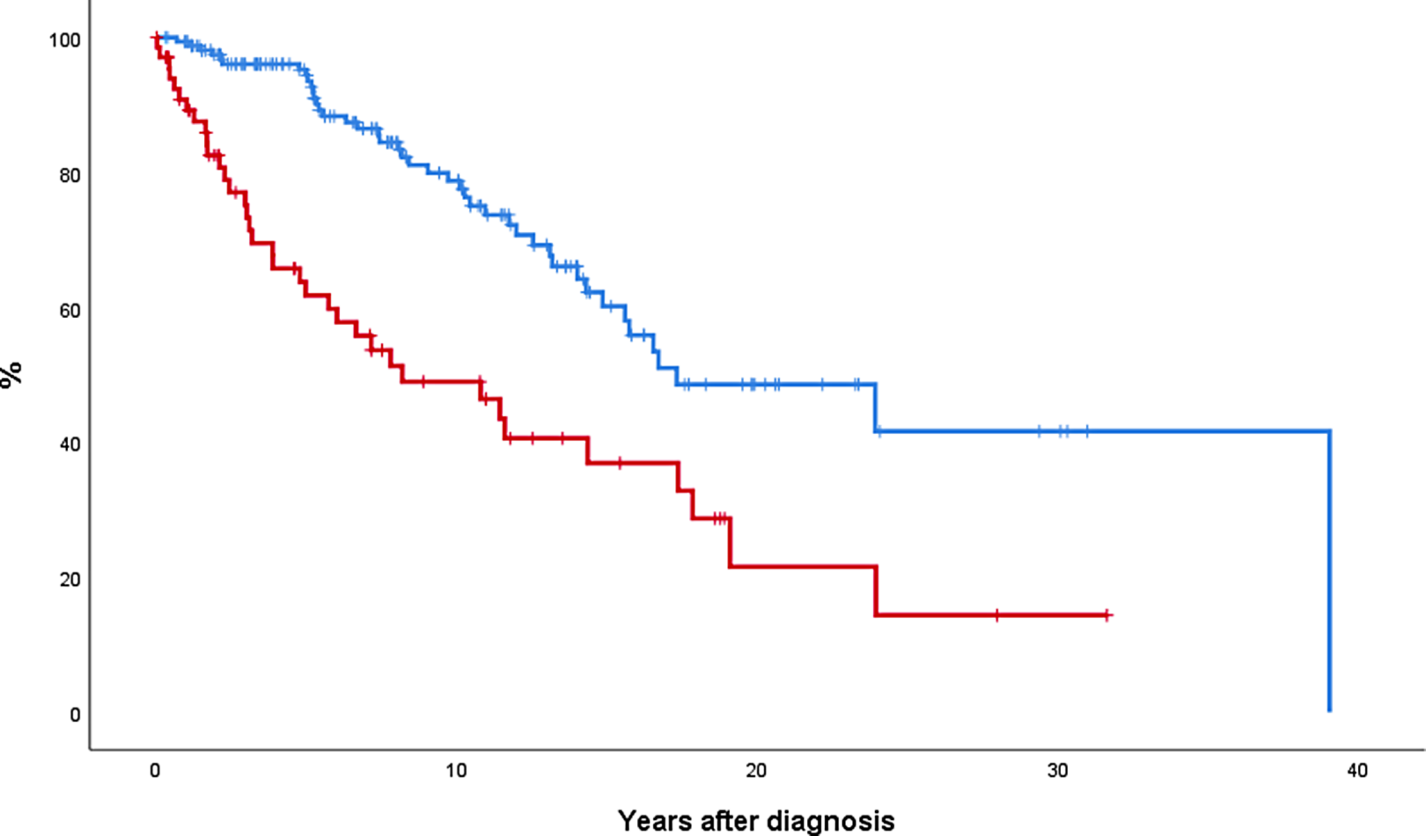
Event-free survival in 69 MPN patients presenting with SVT (red line) and 165 age- and sex-matched patients without SVT at diagnosis (blue line). Median EFS was 8 and 17 years for patients presenting with SVT and those without SVT at diagnosis, respectively (*p* < 0.001). Event-free survival included death, thrombosis, major bleeding or disease progression to myelofibrosis/acute leukemia, whichever occurred in the first place. *Abbreviation*: MPN, myeloproliferative neoplasm; SVT, splanchnic vein thrombosis

Event-free survival (EFS) according to the presence of SVT at MPN diagnosis is shown in Fig. 4. Median EFS was 8 and 17 years in SVT-presenting and non-SVT groups, respectively (*p* < 0.001). Male sex and molecular high-risk were also associated with significant shorter EFS (data not shown). At multivariate analysis, MPN patients presenting with SVT showed a higher risk of presenting any event (HR 3.0, 95% CI 1.9–4.8, *p* < 0.001), adjusted by age (HR 1.04, 95% CI 1.02–1.05, *p* < 0.001), sex (*p* not significant), and molecular risk (*p* not significant).

### Thrombosis and bleeding

In order to avoid bias by selection, patients developing SVT after diagnosis were excluded from thrombosis/bleeding analysis; MPN with previous/presenting SVT (*n* = 69) and MPN controls (*n* = 165) were compared. During follow-up, 36 patients presented at least one thrombotic event resulting in a 10-year probability of thrombosis of 13%. The probability of thrombosis at 10 years in MPN patients presenting with SVT and in age- and sex-matched MPN without SVT was 27% and 8%, respectively (*p* = 0.001). Sex, cardiovascular risk factors, molecular classification and DTA mutations were not associated with higher risk of thrombosis. At multivariate analysis, MPN presenting with SVT showed a higher risk of total thrombosis (HR 3.2, 95% CI 1.6–6.4, *p* = 0.001), adjusted by age (HR 1.03, 95%CI: 0.99–1.06, p = 0.07), sex (*p* not significant), presence of cardiovascular risk factors (*p* not significant) and molecular high-risk (*p* not significant).

A total of 15 arterial thrombotic events were registered resulting in a 10-year probability of arterial thrombosis of 4.9%. Male sex, cardiovascular risk factors and DTA mutations were associated with a significantly higher probability of arterial thrombosis, whereas Grinfeld classification and SVT at diagnosis were not. At multivariate analysis, DTA mutations were associated with a higher risk of arterial thrombosis (HR, 2.9; 95%CI, 1.1–8.7; *p* = 0.049) after correction by age (HR, 1.04; 95%CI, 0.99–1.08; *p* = 0.07) and presence of cardiovascular risk factors (HR, 4.0; 95%CI, 0.86–18.9; *p* = 0.08). The higher probability of arterial thrombosis observed in patients carrying DTA mutation was restricted to the MPN control group since none of the 9 MPN patients presenting with SVT who carried DTA mutations experienced arterial thrombosis during follow-up.

Regarding venous thrombosis, 23 events were reported resulting in a probability of venous thrombosis at 10 years of 9%. Six of these events occurred in the control group and 17 in the SVT group. Among the latter, 3 events were attributed to deep vein thrombosis/pulmonary embolism (DVT/PE), while 14 were instances of SVT progression or SVT re-thrombosis. As expected, the probability of venous thrombosis was significantly different among groups (28% and 1.6% in MPN presenting with SVT and age- and sex-matched MPN controls, respectively, *p* < 0.0001). When the types of venous thrombosis were analyzed separately in MPN presenting with SVT group, the 10-year probabilities of DVT/PE and SVT were 4.4% and 23.6%, respectively. Although we identified a higher probability of developing DVT/PE during the evolution in the SVT group (10-year probability of 1.6% vs. 4.4% in the control vs. SVT group, respectively), this difference did not reach statistical significance.

In patients presenting with SVT, the probability of a new thrombotic event of any type was significantly different according to molecular classification (22% and 64% at 10 years in molecular low- and high-risk, respectively, *p* = 0.01), whereas sex and DTA mutations were not associated with thrombosis. At multivariate analysis restricted to MPN patients presenting with SVT, molecular high-risk was associated with increased risk of a new thrombotic event of any type (HR 5.8, 95% CI 1.4–24.0, *p* = 0.01) after adjustment by age (*p* not significant) and *JAK2* allele burden (*p* not significant).

Major bleeding was registered in 28 patients. The probability of major bleeding at 10 years in MPN patients presenting with SVT and in age- and sex MPN matched controls without SVT was 27% and 3.3%, respectively (*p* < 0.0001). Anti-platelet therapy and anticoagulation were also associated with higher probability of major bleeding, while molecular classification and DTA mutations were not (data not shown).

### Cytoreductive and antithrombotic treatment

With reference to cytoreductive therapy, 72.5% received hydroxyurea; 13.7%, anagrelide; 11.8%, interferon; 9.4%, JAK inhibitors; 3.9%, P32; and 2% busulfan. The proportion of patients that received each treatment was very similar between those who presented SVT previous or at diagnosis and those who did not develop SVT. None of the minimal differences observed were statistically significant (Supplemental Table S3). Specifically, 114 of 165 patients in the control group (69.1%) received hydroxyurea and 52 out of 69 received this drug in the SVT group (75.4%) (*p* = 0.43).

On the other side, antithrombotic treatment was significantly different between control and SVT group, in favor of anti-platelet treatment in the control group and anticoagulant treatment in the SVT group. 88.5% of non-SVT control group received anti-platelet treatment, in contrast to 20.3% who received this therapy in SVT group (*p* < 0.001%). Moreover, only 10.3% of controls received anticoagulant therapy unlike SVT group, in which 92.8% of patients received long-term anticoagulant treatment (*p* < 0.001%).

The proportion of patients that received each therapy was also evaluated within SVT group, according to the molecular risk (heterozygous *JAK2* mutation vs. molecular high-risk) and no significant differences were observed between both subgroups in respect of cytoreductive therapy. In relation to antithrombotic treatment, the proportion of patients with anticoagulant drugs was similar between groups (93.3% vs. 88.9%, *p* = 0.5, in heterozygous *JAK2* and molecular high-risk groups, respectively). Nevertheless, a considerably higher proportion of patients received anti-platelet therapy in the molecular high-risk group (15% vs. 55.9%, *p* = 0.01, in heterozygous *JAK2* and molecular high-risk groups, respectively).

### Disease progression and second neoplasia

During follow-up, 25 out of 255 patients presented disease progression (MF, *n* = 20; AML, *n* = 6). The 10- and 20-year probability of disease progression was 5% and 23.5%, respectively, and it was similar in non-SVT group and SVT-presenting group.

**Fig. 5:**
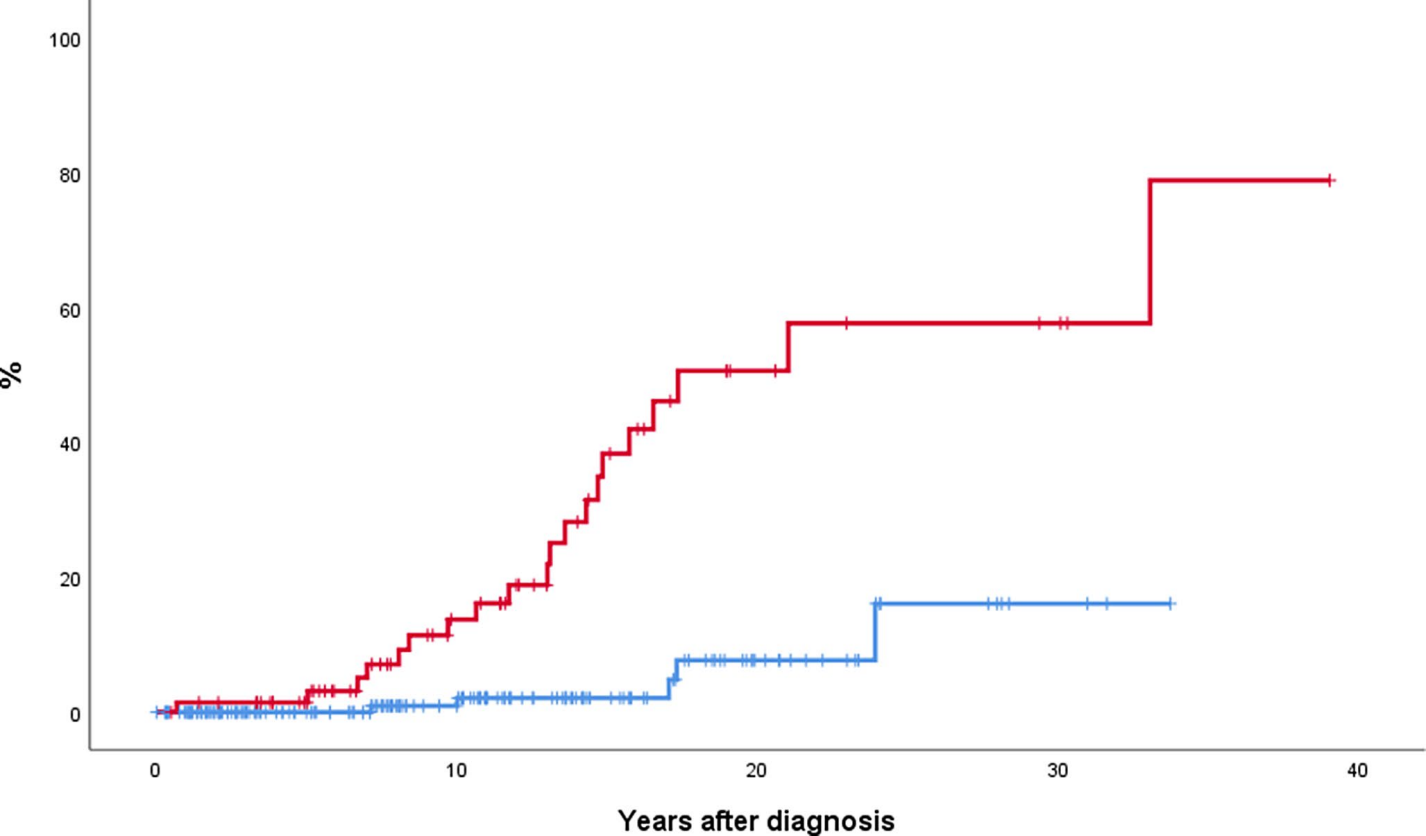
Disease progression according to molecular risk in 255 MPN patients. Kaplan-Meier estimates of disease progression at 20 years was 51% and 8% in patients classified as high and low molecular risk, respectively (p < 0.0001). Molecular high-risk (red line) included patients with *TP53*, chromatin/spliceosome, or homozygous *JAK2* mutation (*n* = 71). Molecular low-risk (blue line) included patients with heterozygous *JAK2* mutation (n = 184). *Abbreviation*: MPN, myeloproliferative neoplasm; SVT, splanchnic vein thrombosis

There were significant differences on time to progression according to molecular classification (20-year probability of disease progression was 44%, 47%, 61%, and 8% in MPN with *TP53* disruption, MPN with chromatin or spliceosome mutation, MPN with homozygous *JAK2* mutation and MPN with heterozygous *JAK2* mutation, respectively, *p* < 0.001). Most events occurred after more than 10 years from diagnosis, except for MPN patients with homozygous *JAK2* mutation in whom disease progression, mainly to MF, was observed widely after 5 years. Time to disease progression is shown in Fig. 5. At multivariate analysis, adjusting by sex, age and SVT, the impact of molecular classification remained significant in molecular high-risk patients (HR, 7.6; 95%CI, 2.5–23.2; *p* < 0.001). DTA mutations did not have a significant impact on time to disease progression.

During follow-up, 33 second neoplasia were reported. There were not significant differences regarding time to second neoplasia among MPN patients presenting with SVT and controls (data not shown).

## Discussion

Herein we describe a series of patients with MPN (PV/ET/MPN-U) and SVT, which have been analyzed separately according to SVT chronology. To our knowledge, this is one of the largest series of PV and ET patients with SVT that have been published so far, including genomic profiling. In addition, a clinical and genomic comparison with an age- and sex-matched group of patients with the same diagnosis but without SVT has been carried out. We chose this control group to underscore the demographic peculiarities of MPN patients presenting with SVT.

We have observed that patients with PV/ET presenting with SVT have a lower *JAK2* allele burden and a lower number of pathogenic mutations than matched MPN patients without SVT of the same age, sex, and diagnosis. This lower molecular complexity might explain the milder hematological picture commonly observed in this group of patients [4, 5, 11]. It has been reported that a proportion of MPN patients presenting with SVT show normal blood counts at MPN diagnosis [4, 7, 12]. In our experience, 19% of patients presenting with SVT were classified as MPN-U, mainly due to absence of erythrocytosis or thrombocytosis. Although hypervolemia secondary to hepatic disease and/or hypersplenism might explain the normalization of blood values, the presence of a latent MPN in which a small *JAK2*-positive clone without additional mutations that has not yet been able to develop an overt MPN would be an alternative explanation. In supporting this, a recent publication showed that acquisition of *JAK2* mutation may precede the appearance of the MPN by decades. In addition, *JAK2*-mutated clonal hematopoiesis of indeterminate potential has been associated with an increased risk of thrombosis [13, 14].

When the Grinfeld algorithm was applied, 87% of patients presenting with SVT corresponded to MPN with heterozygous *JAK2* mutation, a molecular group that is associated with a better survival and a lower rate of disease progression. However, despite belonging mainly to a low-risk molecular group, patients presenting with SVT have a three-fold increased risk of death and a worse event-free survival than controls after correction by age, sex and molecular risk. Prognosis in MPN without SVT is mainly related to MPN subtype, with mortality being associated to MPN progression, especially in MF [6, 15, 16]. We have found that, in PV/ET presenting with SVT, outcome is mostly determined by complications other than MPN progression, especially venous thrombosis, major bleeding and hepatic disease, resulting in a 15-year reduction of median survival.

It is widely known that patients with SVT are at very high risk of recurrent thrombosis despite cytoreductive therapy and anticoagulation [3, 4, 6, 17]. Our work confirms that SVT-presenting MPN has an 8-fold risk of thrombosis recurrence compared with non-SVT MPN, and this risk is even higher when patients belong to a molecular high-risk group. This marked increase risk of thrombosis is present even though there were no significant differences in cytoreductive therapy between groups and a great majority of SVT patients were under anticoagulant therapy.

In addition, we identified the presence of DTA mutations as an independent risk factor for arterial thrombosis in this large cohort of young MPN patients, although the rate of arterial thrombosis in patients presenting with SVT and controls was similar [14, 18]. Finally, NGS profiling showed its highest fitness in predicting disease transformation, confirming previous observations [7, 10]. All these findings reinforce the relevance of NGS-based mutational profiling in young PV/ET patients and, specifically, in complex cases, such as those presenting with SVT, in whom molecular characterization can lead to identification of patients at higher risk of arterial thrombosis, venous re-thrombosis and disease progression. Genomic information besides driver mutation might be incorporated in therapeutic decisions in the future, especially when balance between thrombosis and bleeding is challenging or young patients at high risk of disease progression.

Regarding patients with PV/ET who developed SVT during follow-up, the clinical and hematological characteristics were superimposable with those without SVT, except for a higher frequency of splenomegaly. Interestingly, patients developing SVT during follow-up showed the highest *JAK2* allele burden and the highest number of additional mutations, resulting in a significantly higher proportion of patients belonging to molecular high-risk categories. These findings reinforce the relevance of separating SVT according to its chronology, as they constitute entities with different genomic backgrounds. This heterogeneity affecting MPN with SVT might explain some of the differences with respect to other studies.

Debureaux et al. [7] reported that 29% of 80 patients with SVT were classified as high-risk due to the presence of either *JAK2*V617F allele burden ≥ 50% or chromatin/spliceosome or *TP53* mutations, including 5 patients with MF, 4 patients with *CALR* mutation and 7 cases in whom SVT had appeared during follow-up. In our work, restricted to *JAK2*-mutated patients and excluding MF, molecular high-risk was observed in 13% of patients presenting with SVT and in 52% of those developing SVT during follow-up. Despite these differences in design, both studies showed that risk stratification according to molecular classification is highly useful for disease progression prediction in young patients with PV and ET.

The main limitations of our work are its retrospective design, lack of complete cytogenetic information in most patients and that very rarely mutated genes that have prognostic implications are not included in the NGS panel that we have used. The number of patients included with SVT is limited but this is mainly due to the rarity of both MPN and SVT.

In conclusion, PV and ET presenting with SVT mostly correspond to MPN with heterozygous *JAK2* mutation according to Grinfeld algorithm. Despite this low molecular complexity, MPN presenting with SVT have a shorter survival and event-free survival when compared with PV and ET of similar age and sex without SVT. In young PV/ET patients, the presence of either homozygous *JAK2* mutation or chromatin/spliceosome/*TP53* mutations is associated with a higher risk of disease progression, whereas DTA mutations predict arterial thrombosis. Finally, molecular high-risk predicts development of new thrombotic events in PV/ET patients presenting with SVT.

### Electronic supplementary material

Below is the link to the electronic supplementary material.

Supplementary Material 1

## Data Availability

Data that has been generated during this research project is available from the corresponding author upon reasonable request. Additional information can be found in Supplemental data.

## Abbreviations

VAF: variant allele frequency
MPN: myeloproliferative neoplasm
SVT: splanchnic vein thrombosis
