# Supplementary material for: Genomic classification and outcomes of young patients with polycythemia vera and essential thrombocythemia according to the presence of splanchnic vein thrombosis and its chronology
Source: Ann Hematol. 2024 Jan 24;103(3):737–47. doi: 10.1007/s00277-023-05610-x (PMC10866782; doi:10.1007/s00277-023-05610-x)
Supplement: Supplementary file 1 — Supplementary Material 1 [file 277_2023_5610_MOESM1_ESM.docx]

**Supplemental Table S1: Genes included in the Next-Generation Sequencing panel Myeloid Solution by Sophia Genetics (customized) and their coverage**

| **Gene** | **Coverage** |
| --- | --- |
| ***ABL1*** | Exons 4-9 |
| ***ASXL1*** | Exons 9, 11, and 12 |
| ***BRAF*** | Exon 15 |
| ***CALR*** | Exon 9 |
| ***CBL*** | Exons 8 and 9 |
| ***CEBPA*** | Complete |
| ***CSF3R*** | Complete |
| ***CSNK1A1*** | Exons 3 and 4 |
| ***DNMT3A*** | Complete |
| ***ETV6*** | Complete |
| ***EZH2*** | Complete |
| ***FLT3*** | Exons 13, 15 and 20 |
| ***HRAS*** | Exons 2 and 3 |
| ***IDH1*** | Exon 4 |
| ***IDH2*** | Exon 4 |
| ***JAK2*** | Complete |
| ***KIT*** | Exons 2, 8-11, 13, 17 and 18 |
| ***KMT2A*** | Exons 1-9 and 27 |
| ***KRAS*** | Exons 2 and 3 |
| ***MPL*** | Complete |
| ***NPM1*** | Exons 10 and 11 |
| ***NRAS*** | Exons 2 and 3 |
| ***PTPN11*** | Exons 3, 7-13 |
| ***RUNX1*** | Complete |
| ***SETBP1*** | Exon 4 |
| ***SF3B1*** | Exons 14-16 |
| ***SRSF2*** | Exon 1 |
| ***TET2*** | Complete |
| ***TP53*** | Complete |
| ***U2AF1*** | Exons 2 and 6 |
| ***WT1*** | Exons 6 and 10 |
| ***ZRSR2*** | Complete |

**Supplemental Table S2: Pathogenic and likely pathogenic variants found in our analysis, including allele frequency and manual categorization**

| **Gene** | **DNA** | **Protein** | **Allele frequency** | **Mutation type** | **Pathogenicity class** |
| --- | --- | --- | --- | --- | --- |
| ***ASXL1*** | c.1544_1545del | p.(Val515Glyfs*13) | 30,0 | Frameshift | Pathogenic |
| ***ASXL1*** | c.1935_1937dupG | p.(Gly646Trpfs*12) | 27,7 | Frameshift | Pathogenic |
| ***ASXL1*** | c.1438G>T | p.(Glu480*) | 46,5 | Nonsense | Pathogenic |
| ***ASXL1*** | c.1934dupG | p.(Gly646Trpfs*12) | 20,1 | Frameshift | Pathogenic |
| ***ASXL1*** | c.1549C>T | p.(Gln517*) | 13,6 | Nonsense | Pathogenic |
| ***ASXL1*** | c.1934dupG | p.(Gly646Trpfs*12) | 16,2 | Frameshift | Pathogenic |
| ***ASXL1*** | c.2385del | p.(Trp796Glyfs*22) | 31,0 | Frameshift | Pathogenic |
| ***ASXL1*** | c.1900_1922del | p.(Glu635Argfs*15) | 10,0 | Frameshift | Pathogenic |
| ***ASXL1*** | c.2348del | p.(Pro783Argfs*35) | 15,9 | Frameshift | Likely pathogenic |
| ***ASXL1*** | c.1774C>T | p.(Gln592*) | 32,6 | Nonsense | Likely pathogenic |
| ***ASXL1*** | c.1786dupC | p.(Arg596Profs*23) | 5,7 | Frameshift | Likely pathogenic |
| ***ASXL1*** | c.2138del | p.(Met713Serfs*12) | 42,9 | Frameshift | Likely pathogenic |
| ***ASXL1*** | c.2125_2129del | p.(Ala709Asnfs*7) | 12,4 | Frameshift | Likely pathogenic |
| ***ASXL1*** | c.2128G>T | p.(Gly710*) | 11,8 | Nonsense | Likely pathogenic |
| ***ASXL1*** | c.2465_2469delCATTA | p.(Thr822ArsfsTer9) | 31,7 | Nonsense | Likely pathogenic |
| ***ASXL1*** | c.3105del | p.(Asn1036Ilefs*11) | 3,7 | Frameshift | Likely pathogenic |
| ***ASXL1*** | c.1934dupG | p.(Gly646Trpfs*12) | 28,2 | Frameshift | Likely pathogenic |
| ***CALR*** | c.1103_1136del | p.(Lys368Argfs*?) | 38,4 | Frameshift | Pathogenic |
| ***CALR*** | c.1103del | p.(Lys368Argfs*?) | 6,1 | Frameshift | Pathogenic |
| ***CALR*** | c.1154_1155insTTGTC | p.(Lys385Asnfs*?) | 20,6 | Frameshift | Pathogenic |
| ***CALR*** | c.1099_1150del | p.(Leu367Thrfs*?) | 42,0 | Frameshift | Pathogenic |
| ***CALR*** | c.1119_1123del | p.(Asp373Glufs*14) | 31,0 | Frameshift | Pathogenic |
| ***CALR*** | c.1128_1141del | p.(Lys377Glyfs*7) | 31,0 | Frameshift | Pathogenic |
| ***CALR*** | c.1112A>C | p.(Glu371Ala) | 4,7 | Missense | Likely pathogenic |
| ***CBL*** | c.1151G>A | p.(Cys384Tyr) | 45,8 | Missense | Likely pathogenic |
| ***CBL*** | c.1253T>C | p.(Phe418Ser) | 2,4 | Missense | Likely pathogenic |
| ***DNMT3A*** | c.2644C>T | p.(Arg882Cys) | 15,7 | Missense | Pathogenic |
| ***DNMT3A*** | c.2645G>A | p.(Arg882His) | 42,2 | Missense | Pathogenic |
| ***DNMT3A*** | c.2510C>G | p.(Ser837*) | 31,5 | Nonsense | Likely pathogenic |
| ***DNMT3A*** | c.2645G>C | p.(Arg882Pro) | 41,9 | Missense | Likely pathogenic |
| ***DNMT3A*** | c.1672_1677delTTTTGC | p.(Phe558_Cys559del) | 36,1 | Frameshift | Likely pathogenic |
| ***DNMT3A*** | c.2186G>A | p.(Arg729Gln) | 4,0 | Missense | Likely pathogenic |
| ***DNMT3A*** | c.2186G>A | p.(Arg729Gln) | 8,2 | Missense | Likely pathogenic |
| ***DNMT3A*** | c.2264T>C | p.(Phe755Ser) | 24,9 | Missense | Likely pathogenic |
| ***DNMT3A*** | c.2071dupA | p.(Thr691Asnfs*22) | 6,6 | Frameshift | Likely pathogenic |
| ***DNMT3A*** | c.1975dupC | p.(Arg659Profs*9) | 18,0 | Frameshift | Likely pathogenic |
| ***DNMT3A*** | c.2309C>T | p.(Ser770Leu) | 34,5 | Missense | Likely pathogenic |
| ***DNMT3A*** | c.2711C>T | p.(Pro904Leu) | 4,8 | Missense | Likely pathogenic |
| ***DNMT3A*** | c.1903C>T | p.(Arg635Trp) | 45,4 | Missense | Likely pathogenic |
| ***DNMT3A*** | c.1937-2A>G |  | 3,0 | Splice acceptor -2 | Likely pathogenic |
| ***DNMT3A*** | c.2309C>T | p.(Ser770Leu) | 3,9 | Missense | Likely pathogenic |
| ***ETV6*** | c.190G>A | p.(Asp64Asn) | 51,2 | Missense | Pathogenic |
| ***EZH2*** | c.392T>A | p.(Ile131Asn) | 11,0 | Missense | Likely pathogenic |
| ***IDH1*** | c.395G>A | p.(Arg132His) | 8,8 | Missense | Pathogenic |
| ***IDH1*** | c.394C>T | p.(Arg132Cys) | 26,5 | Missense | Likely pathogenic |
| ***IDH2*** | c.419G>A | p.(Arg140Gln) | 46,5 | Missense | Pathogenic |
| ***IDH2*** | c.419G>A | p.(Arg140Gln) | 40,9 | Missense | Pathogenic |
| ***JAK2*** | c.1849G>T | p.(Val617Phe) | 32,8 | Missense | Pathogenic |
| ***JAK2*** | c.1849G>T | p.(Val617Phe) | 12,6 | Missense | Pathogenic |
| ***JAK2*** | c.1849G>T | p.(Val617Phe) | 36,8 | Missense | Pathogenic |
| ***JAK2*** | c.1849G>T | p.(Val617Phe) | 34,4 | Missense | Pathogenic |
| ***JAK2*** | c.1849G>T | p.(Val617Phe) | 51,1 | Missense | Pathogenic |
| ***JAK2*** | c.1849G>T | p.(Val617Phe) | 20,8 | Missense | Pathogenic |
| ***JAK2*** | c.1849G>T | p.(Val617Phe) | 31,7 |  | Pathogenic |
| ***JAK2*** | c.1849G>T | p.(Val617Phe) | 10,7 | Missense | Pathogenic |
| ***JAK2*** | c.1849G>T | p.(Val617Phe) | 2,8 | Missense | Pathogenic |
| ***JAK2*** | c.1849G>T | p.(Val617Phe) | 55,1 | Missense | Pathogenic |
| ***JAK2*** | c.1849G>T | p.(Val617Phe) | 9,4 | Missense | Pathogenic |
| ***JAK2*** | c.1849G>T | p.(Val617Phe) | 42,2 | Missense | Pathogenic |
| ***JAK2*** | c.1849G>T | p.(Val617Phe) | 18,4 | Missense | Pathogenic |
| ***JAK2*** | c.1849G>T | p.(Val617Phe) | 30,1 | Missense | Pathogenic |
| ***JAK2*** | c.1849G>T | p.(Val617Phe) | 23,0 | Missense | Pathogenic |
| ***JAK2*** | c.1849G>T | p.(Val617Phe) | 41,0 | Missense | Pathogenic |
| ***JAK2*** | c.1849G>T | p.(Val617Phe) | 21,1 | Missense | Pathogenic |
| ***JAK2*** | c.1849G>T | p.(Val617Phe) | 23,6 | Missense | Pathogenic |
| ***JAK2*** | c.1849G>T | p.(Val617Phe) | 28,0 | Missense | Pathogenic |
| ***JAK2*** | c.1849G>T | p.(Val617Phe) | 73,1 | Missense | Pathogenic |
| ***JAK2*** | c.1849G>T | p.(Val617Phe) | 11,3 | Missense | Pathogenic |
| ***JAK2*** | c.1849G>T | p.(Val617Phe) | 32,4 | Missense | Pathogenic |
| ***JAK2*** | c.1849G>T | p.(Val617Phe) | 36,4 | Missense | Pathogenic |
| ***JAK2*** | c.1849G>T | p.(Val617Phe) | 44,8 | Missense | Pathogenic |
| ***JAK2*** | c.1849G>T | p.(Val617Phe) | 25,8 | Missense | Pathogenic |
| ***JAK2*** | c.1849G>T | p.(Val617Phe) | 7,7 | Missense | Pathogenic |
| ***JAK2*** | c.1849G>T | p.(Val617Phe) | 21,1 | Missense | Pathogenic |
| ***JAK2*** | c.1849G>T | p.(Val617Phe) | 17,4 | Missense | Pathogenic |
| ***JAK2*** | c.1849G>T | p.(Val617Phe) | 16,4 | Missense | Pathogenic |
| ***JAK2*** | c.1849G>T | p.(Val617Phe) | 21,2 | Missense | Pathogenic |
| ***JAK2*** | c.1849G>T | p.(Val617Phe) | 57,9 | Missense | Pathogenic |
| ***JAK2*** | c.1849G>T | p.(Val617Phe) | 19,9 | Missense | Pathogenic |
| ***JAK2*** | c.1849G>T | p.(Val617Phe) | 30,9 | Missense | Pathogenic |
| ***JAK2*** | c.1849G>T | p.(Val617Phe) | 36,0 | Missense | Pathogenic |
| ***JAK2*** | c.1849G>T | p.(Val617Phe) | 35,9 | Missense | Pathogenic |
| ***JAK2*** | c.1849G>T | p.(Val617Phe) | 31,4 | Missense | Pathogenic |
| ***JAK2*** | c.1849G>T | p.(Val617Phe) | 26,4 | Missense | Pathogenic |
| ***JAK2*** | c.1849G>T | p.(Val617Phe) | 10,0 | Missense | Pathogenic |
| ***JAK2*** | c. 1849G>T | p.(Val617Phe) | 23,9 | Missense | Pathogenic |
| ***JAK2*** | c.1849G>T | p.(Val617Phe) | 35,1 | Missense | Pathogenic |
| ***JAK2*** | c.1849G>T | p.(Val617Phe) | 10,0 | Missense | Pathogenic |
| ***JAK2*** | c.1849G>T | p.(Val617Phe) | 8,9 | Missense | Pathogenic |
| ***JAK2*** | c.1849G>T | p.(Val617Phe) | 26,5 | Missense | Pathogenic |
| ***JAK2*** | c.1849G>T | p.(Val617Phe) | 19,6 | Missense | Pathogenic |
| ***JAK2*** | c.1849G>T | p.(Val617Phe) | 31,0 | Missense | Pathogenic |
| ***JAK2*** | c.1849G>T | p.(Val617Phe) | 42,1 | Missense | Pathogenic |
| ***JAK2*** | c.1849G>T | p.(Val617Phe) | 51,8 | Missense | Pathogenic |
| ***JAK2*** | c.1849G>T | p.(Val617Phe) | 11,0 | Missense | Pathogenic |
| ***JAK2*** | c.1849G>T | p.(Val617Phe) | 29,5 | Missense | Pathogenic |
| ***JAK2*** | c.1849G>T | p.(Val617Phe) | 21,5 | Missense | Pathogenic |
| ***JAK2*** | c.1849G>T | p.(Val617Phe) | 45,7 | Missense | Pathogenic |
| ***JAK2*** | c.1849G>T | p.(Val617Phe) | 6,1 | Missense | Pathogenic |
| ***JAK2*** | c.1849G>T | p.(Val617Phe) | 17,1 | Missense | Pathogenic |
| ***JAK2*** | c.1849G>T | p.(Val617Phe) | 42,8 | Missense | Pathogenic |
| ***JAK2*** | c.1849G>T | p.(Val617Phe) | 38,7 | Missense | Pathogenic |
| ***JAK2*** | c.1849G>T | p.(Val617Phe) | 26,0 | Missense | Pathogenic |
| ***JAK2*** | c.1849G>T | p.(Val617Phe) | 9,5 | Missense | Pathogenic |
| ***JAK2*** | c.1849G>T | p.(Val617Phe) | 37,8 | Missense | Pathogenic |
| ***JAK2*** | c.1849G>T | p.(Val617Phe) | 16,4 | Missense | Pathogenic |
| ***JAK2*** | c.1849G>T | p.(Val617Phe) | 16,3 | Missense | Pathogenic |
| ***JAK2*** | c.1849G>T | p.(Val617Phe) | 23,3 | Missense | Pathogenic |
| ***JAK2*** | c.1849G>T | p.(Val617Phe) | 11,5 | Missense | Pathogenic |
| ***JAK2*** | c.1849G>T | p.(Val617Phe) | 25,2 | Missense | Pathogenic |
| ***JAK2*** | c.1849G>T | p.(Val617Phe) | 29,1 | Missense | Pathogenic |
| ***JAK2*** | c.1849G>T | p.(Val617Phe) | 38,8 | Missense | Pathogenic |
| ***JAK2*** | c.1849G>T | p.(Val617Phe) | 8,5 | Missense | Pathogenic |
| ***JAK2*** | c.1849G>T | p.(Val617Phe) | 46,9 | Missense | Pathogenic |
| ***JAK2*** | c.1849G>T | p.(Val617Phe) | 26,1 | Missense | Pathogenic |
| ***JAK2*** | c.1849G>T | p.(Val617Phe) | 17,2 | Missense | Pathogenic |
| ***JAK2*** | c.1849G>T | p.(Val617Phe) | 10,5 | Missense | Pathogenic |
| ***JAK2*** | c.1849G>T | p.(Val617Phe) | 61,2 | Missense | Pathogenic |
| ***JAK2*** | c.1849G>T | p.(Val617Phe) | 39,2 | Missense | Pathogenic |
| ***JAK2*** | c.1849G>T | p.(Val617Phe) | 8,4 | Missense | Pathogenic |
| ***JAK2*** | c.1849G>T | p.(Val617Phe) | 60,6 | Missense | Pathogenic |
| ***JAK2*** | c.1849G>T | p.(Val617Phe) | 15,2 | Missense | Pathogenic |
| ***JAK2*** | c.1849G>T | p.(Val617Phe) | 15,4 | Missense | Pathogenic |
| ***JAK2*** | c.1849G>T | p.(Val617Phe) | 61,9 | Missense | Pathogenic |
| ***JAK2*** | c.1849G>T | p.(Val617Phe) | 16,9 | Missense | Pathogenic |
| ***JAK2*** | c.1849G>T | p.(Val617Phe) | 8,4 | Missense | Pathogenic |
| ***JAK2*** | c.1849G>T | p.(Val617Phe) | 14,4 | Missense | Pathogenic |
| ***JAK2*** | c.1849G>T | p.(Val617Phe) | 81,4 | Missense | Pathogenic |
| ***JAK2*** | c.1849G>T | p.(Val617Phe) | 44,6 | Missense | Pathogenic |
| ***JAK2*** | c.1849G>T | p.(Val617Phe) | 15,9 | Missense | Pathogenic |
| ***JAK2*** | c.1849G>T | p.(Val617Phe) | 99,3 | Missense | Pathogenic |
| ***JAK2*** | c.1849G>T | p.(Val617Phe) | 44,1 | Missense | Pathogenic |
| ***JAK2*** | c.1849G>T | p.(Val617Phe) | 30,1 | Missense | Pathogenic |
| ***JAK2*** | c.1849G>T | p.(Val617Phe) | 68,8 | Missense | Pathogenic |
| ***JAK2*** | c.1849G>T | p.(Val617Phe) | 35,4 | Missense | Pathogenic |
| ***JAK2*** | c.1849G>T | p.(Val617Phe) | 18,3 | Missense | Pathogenic |
| ***JAK2*** | c.1849G>T | p.(Val617Phe) | 23,0 | Missense | Pathogenic |
| ***JAK2*** | c.1849G>T | p.(Val617Phe) | 73,7 | Missense | Pathogenic |
| ***JAK2*** | c.1849G>T | p.(Val617Phe) | 21,3 | Missense | Pathogenic |
| ***JAK2*** | c.1849G>T | p.(Val617Phe) | 53,3 | Missense | Pathogenic |
| ***JAK2*** | c.1849G>T | p.(Val617Phe) | 97,5 | Missense | Pathogenic |
| ***JAK2*** | c.1849G>T | p.(Val617Phe) | 10,0 | Missense | Pathogenic |
| ***JAK2*** | c.1849G>T | p.(Val617Phe) | 60,5 | Missense | Pathogenic |
| ***JAK2*** | c.1849G>T | p.(Val617Phe) | 72,2 | Missense | Pathogenic |
| ***JAK2*** | c.1849G>T | p.(Val617Phe) | 24,0 | Missense | Pathogenic |
| ***JAK2*** | c.1849G>T | p.(Val617Phe) | 24,5 | Missense | Pathogenic |
| ***JAK2*** | c.1849G>T | p.(Val617Phe) | 68,2 | Missense | Pathogenic |
| ***JAK2*** | c.1849G>T | p.(Val617Phe) | 20,7 | Missense | Pathogenic |
| ***JAK2*** | c.1849G>T | p.(Val617Phe) | 3,0 | Missense | Pathogenic |
| ***JAK2*** | c.1849G>T | p.(Val617Phe) | 6,8 | Missense | Pathogenic |
| ***JAK2*** | c.1849G>T | p.(Val617Phe) | 25,5 | Missense | Pathogenic |
| ***JAK2*** | c.1849G>T | p.(Val617Phe) | 10,1 | Missense | Pathogenic |
| ***JAK2*** | c.1849G>T | p.(Val617Phe) | 11,6 | Missense | Pathogenic |
| ***JAK2*** | c.1849G>T | p.(Val617Phe) | 3,1 | Missense | Pathogenic |
| ***JAK2*** | c.1849G>T | p.(Val617Phe) | 19,8 | Missense | Pathogenic |
| ***JAK2*** | c.1849G>T | p.(Val617Phe) | 25,9 | Missense | Pathogenic |
| ***JAK2*** | c.1849G>T | p.(Val617Phe) | 13,9 | Missense | Pathogenic |
| ***JAK2*** | c.1849G>T | p.(Val617Phe) | 18,7 | Missense | Pathogenic |
| ***JAK2*** | c.1849G>T | p.(Val617Phe) | 15,8 | Missense | Pathogenic |
| ***JAK2*** | c.1849G>T | p.(Val617Phe) | 20,5 | Missense | Pathogenic |
| ***JAK2*** | c.1849G>T | p.(Val617Phe) | 23,2 | Missense | Pathogenic |
| ***JAK2*** | c.1849G>T | p.(Val617Phe) | 15,0 | Missense | Pathogenic |
| ***JAK2*** | c.1849G>T | p.(Val617Phe) | 8,9 | Missense | Pathogenic |
| ***JAK2*** | c.1849G>T | p.(Val617Phe) | 29,4 | Missense | Pathogenic |
| ***JAK2*** | c.1849G>T | p.(Val617Phe) | 5,1 | Missense | Pathogenic |
| ***JAK2*** | c.1849G>T | p.(Val617Phe) | 18,9 | Missense | Pathogenic |
| ***JAK2*** | c.1849G>T | p.(Val617Phe) | 2,5 | Missense | Pathogenic |
| ***JAK2*** | c.1849G>T | p.(Val617Phe) | 27,3 | Missense | Pathogenic |
| ***JAK2*** | c.1849G>T | p.(Val617Phe) | 13,7 | Missense | Pathogenic |
| ***JAK2*** | c.1849G>T | p.(Val617Phe) | 32,3 | Missense | Pathogenic |
| ***JAK2*** | c.1849G>T | p.(Val617Phe) | 11,4 | Missense | Pathogenic |
| ***JAK2*** | c.1849G>T | p.(Val617Phe) | 6,5 | Missense | Pathogenic |
| ***JAK2*** | c.1849G>T | p.(Val617Phe) | 16,7 | Missense | Pathogenic |
| ***JAK2*** | c.1849G>T | p.(Val617Phe) | 36,7 | Missense | Pathogenic |
| ***JAK2*** | c.1849G>T | p.(Val617Phe) | 14,6 | Missense | Pathogenic |
| ***JAK2*** | c.1849G>T | p.(Val617Phe) | 18,9 | Missense | Pathogenic |
| ***JAK2*** | c.1849G>T | p.(Val617Phe) | 21,2 | Missense | Pathogenic |
| ***JAK2*** | c.1849G>T | p.(Val617Phe) | 23,6 | Missense | Pathogenic |
| ***JAK2*** | c.1849G>T | p.(Val617Phe) | 8,6 | Missense | Pathogenic |
| ***JAK2*** | c.1849G>T | p.(Val617Phe) | 19,0 | Missense | Pathogenic |
| ***JAK2*** | c.1849G>T | p.(Val617Phe) | 8,9 | Missense | Pathogenic |
| ***JAK2*** | c.1849G>T | p.(Val617Phe) | 26,7 | Missense | Pathogenic |
| ***JAK2*** | c.1849G>T | p.(Val617Phe) | 34,8 | Missense | Pathogenic |
| ***JAK2*** | c.1849G>T | p.(Val617Phe) | 17,0 | Missense | Pathogenic |
| ***JAK2*** | c.1849G>T | p.(Val617Phe) | 23,2 | Missense | Pathogenic |
| ***JAK2*** | c.1849G>T | p.(Val617Phe) | 19,6 | Missense | Pathogenic |
| ***JAK2*** | c.1849G>T | p.(Val617Phe) | 19,6 | Missense | Pathogenic |
| ***JAK2*** | c.1849G>T | p.(Val617Phe) | 17,1 | Missense | Pathogenic |
| ***JAK2*** | c.1849G>T | p.(Val617Phe) | 92,7 | Missense | Pathogenic |
| ***JAK2*** | c.1849G>T | p.(Val617Phe) | 40,9 | Missense | Pathogenic |
| ***JAK2*** | c.1849G>T | p.(Val617Phe) | 4,9 | Missense | Pathogenic |
| ***JAK2*** | c.1849G>T | p.(Val617Phe) | 18,7 | Missense | Pathogenic |
| ***JAK2*** | c.1849G>T | p.(Val617Phe) | 16,5 | Missense | Pathogenic |
| ***JAK2*** | c.1849G>T | p.(Val617Phe) | 19,0 | Missense | Pathogenic |
| ***JAK2*** | c.1849G>T | p.(Val617Phe) | 18,7 | Missense | Pathogenic |
| ***JAK2*** | c.1849G>T | p.(Val617Phe) | 16,9 | Missense | Pathogenic |
| ***JAK2*** | c.1849G>T | p.(Val617Phe) | 30,1 | Missense | Pathogenic |
| ***JAK2*** | c.1849G>T | p.(Val617Phe) | 10,0 | Missense | Pathogenic |
| ***JAK2*** | c.1849G>T | p.(Val617Phe) | 21,8 | Missense | Pathogenic |
| ***JAK2*** | c.1849G>T | p.(Val617Phe) | 3,8 | Missense | Pathogenic |
| ***JAK2*** | c.1849G>T | p.(Val617Phe) | 11,6 | Missense | Pathogenic |
| ***JAK2*** | c.1849G>T | p.(Val617Phe) | 16,9 | Missense | Pathogenic |
| ***JAK2*** | c.1849G>T | p.(Val617Phe) | 12,6 | Missense | Pathogenic |
| ***JAK2*** | c.1849G>T | p.(Val617Phe) | 35,3 | Missense | Pathogenic |
| ***JAK2*** | c.1849G>T | p.(Val617Phe) | 11,9 | Missense | Pathogenic |
| ***JAK2*** | c.1849G>T | p.(Val617Phe) | 8,1 | Missense | Pathogenic |
| ***JAK2*** | c.1849G>T | p.(Val617Phe) | 16,4 | Missense | Pathogenic |
| ***JAK2*** | c.1849G>T | p.(Val617Phe) | 11,5 | Missense | Pathogenic |
| ***JAK2*** | c.1849G>T | p.(Val617Phe) | 13,7 | Missense | Pathogenic |
| ***JAK2*** | c.1849G>T | p.(Val617Phe) | 96,2 | Missense | Pathogenic |
| ***JAK2*** | c.1849G>T | p.(Val617Phe) | 56,4 | Missense | Pathogenic |
| ***JAK2*** | c.1849G>T | p.(Val617Phe) | 9,4 | Missense | Pathogenic |
| ***JAK2*** | c.1849G>T | p.(Val617Phe) | 8,3 | Missense | Pathogenic |
| ***JAK2*** | c.1849G>T | p.(Val617Phe) | 3,2 | Missense | Pathogenic |
| ***JAK2*** | c.1849G>T | p.(Val617Phe) | 8,5 | Missense | Pathogenic |
| ***JAK2*** | c.1849G>T | p.(Val617Phe) | 1,9 | Missense | Pathogenic |
| ***JAK2*** | c.1849G>T | p.(Val617Phe) | 6,2 | Missense | Pathogenic |
| ***JAK2*** | c.1849G>T | p.(Val617Phe) | 5,6 | Missense | Pathogenic |
| ***JAK2*** | c.1849G>T | p.(Val617Phe) | 82,3 | Missense | Pathogenic |
| ***JAK2*** | c.1849G>T | p.(Val617Phe) | 16,7 | Missense | Pathogenic |
| ***JAK2*** | c.1849G>T | p.(Val617Phe) | 4,8 | Missense | Pathogenic |
| ***JAK2*** | c.1849G>T | p.(Val617Phe) | 19,8 | Missense | Pathogenic |
| ***JAK2*** | c.1849G>T | p.(Val617Phe) | 12,9 | Missense | Pathogenic |
| ***JAK2*** | c.1849G>T | p.(Val617Phe) | 3,8 | Missense | Pathogenic |
| ***JAK2*** | c.1849G>T | p.(Val617Phe) | 42,3 | Missense | Pathogenic |
| ***JAK2*** | c.1849G>T | p.(Val617Phe) | 72,8 | Missense | Pathogenic |
| ***JAK2*** | c.1849G>T | p.(Val617Phe) | 5,6 | Missense | Pathogenic |
| ***JAK2*** | c.1849G>T | p.(Val617Phe) | 38,6 | Missense | Pathogenic |
| ***JAK2*** | c.1849G>T | p.(Val617Phe) | 41,0 | Missense | Pathogenic |
| ***JAK2*** | c.1849G>T | p.(Val617Phe) | 41,8 | Missense | Pathogenic |
| ***JAK2*** | c.1849G>T | p.(Val617Phe) | 76,1 | Missense | Pathogenic |
| ***JAK2*** | c.1849G>T | p.(Val617Phe) | 24,4 | Missense | Pathogenic |
| ***JAK2*** | c.1849G>T | p.(Val617Phe) | 29,2 | Missense | Pathogenic |
| ***JAK2*** | c.1849G>T | p.(Val617Phe) | 87,5 | Missense | Pathogenic |
| ***JAK2*** | c.1849G>T | p.(Val617Phe) | 90,0 | Missense | Pathogenic |
| ***JAK2*** | c.1849G>T | p.(Val617Phe) | 44,0 | Missense | Pathogenic |
| ***JAK2*** | c.1849G>T | p.(Val617Phe) | 3,6 | Missense | Pathogenic |
| ***JAK2*** | c.1849G>T | p.(Val617Phe) | 23,9 | Missense | Pathogenic |
| ***JAK2*** | c.1849G>T | p.(Val617Phe) | 29,0 | Missense | Pathogenic |
| ***JAK2*** | c.1849G>T | p.(Val617Phe) | 4,8 | Missense | Pathogenic |
| ***JAK2*** | c.1849G>T | p.(Val617Phe) | 24,8 | Missense | Pathogenic |
| ***JAK2*** | c.1849G>T | p.(Val617Phe) | 41,4 | Missense | Pathogenic |
| ***JAK2*** | c.1849G>T | p.(Val617Phe) | 6,4 | Missense | Pathogenic |
| ***JAK2*** | c.1849G>T | p.(Val617Phe) | 14,5 | Missense | Pathogenic |
| ***JAK2*** | c.1849G>T | p.(Val617Phe) | 80,6 | Missense | Pathogenic |
| ***JAK2*** | c.1849G>T | p.(Val617Phe) | 7,8 | Missense | Pathogenic |
| ***JAK2*** | c.1849G>T | p.(Val617Phe) | 5,4 | Missense | Pathogenic |
| ***JAK2*** | c.1849G>T | p.(Val617Phe) | 58,0 | Missense | Pathogenic |
| ***JAK2*** | c.1849G>T | p.(Val617Phe) | 21,5 | Missense | Pathogenic |
| ***JAK2*** | c.1849G>T | p.(Val617Phe) | 73,5 | Missense | Pathogenic |
| ***JAK2*** | c.1849G>T | p.(Val617Phe) | 29,2 | Missense | Pathogenic |
| ***JAK2*** | c.1849G>T | p.(Val617Phe) | 53,1 | Missense | Pathogenic |
| ***JAK2*** | c.1849G>T | p.(Val617Phe) | 65,1 | Missense | Pathogenic |
| ***JAK2*** | c.1849G>T | p.(Val617Phe) | 12,0 | Missense | Pathogenic |
| ***JAK2*** | c.1849G>T | p.(Val617Phe) | 20,0 | Missense | Pathogenic |
| ***JAK2*** | c.1849G>T | p.(Val617Phe) | 29,0 | Missense | Pathogenic |
| ***JAK2*** | c.1849G>T | p.(Val617Phe) | 17,5 | Missense | Pathogenic |
| ***JAK2*** | c.1849G>T | p.(Val617Phe) | 28,6 | Missense | Pathogenic |
| ***JAK2*** | c.1849G>T | p.(Val617Phe) | 45,6 | Missense | Pathogenic |
| ***JAK2*** | c.1849G>T | p.(Val617Phe) | 36,6 | Missense | Pathogenic |
| ***JAK2*** | c.1849G>T | p.(Val617Phe) | 50,2 | Missense | Pathogenic |
| ***JAK2*** | c.1849G>T | p.(Val617Phe) | 26,1 | Missense | Pathogenic |
| ***JAK2*** | c.1849G>T | p.(Val617Phe) | 32,8 | Missense | Pathogenic |
| ***JAK2*** | c.1849G>T | p.(Val617Phe) | 42,8 | Missense | Pathogenic |
| ***JAK2*** | c.1849G>T | p.(Val617Phe) | 14,2 | Missense | Pathogenic |
| ***JAK2*** | c.1849G>T | p.(Val617Phe) | 55,7 | Missense | Pathogenic |
| ***JAK2*** | c.1849G>T | p.(Val617Phe) | 8,6 | Missense | Pathogenic |
| ***JAK2*** | c.1849G>T | p.(Val617Phe) | 54,9 | Missense | Pathogenic |
| ***JAK2*** | c.1849G>T | p.(Val617Phe) | 77,4 | Missense | Pathogenic |
| ***JAK2*** | c.1849G>T | p.(Val617Phe) | 33,8 | Missense | Pathogenic |
| ***JAK2*** | c.1849G>T | p.(Val617Phe) | 94,4 | Missense | Pathogenic |
| ***JAK2*** | c.1849G>T | p.(Val617Phe) | 29,3 | Missense | Pathogenic |
| ***JAK2*** | c.1849G>T | p.(Val617Phe) | 62,4 | Missense | Pathogenic |
| ***JAK2*** | c.1849G>T | p.(Val617Phe) | 41,5 | Missense | Pathogenic |
| ***JAK2*** | c.1849G>T | p.(Val617Phe) | 17,9 | Missense | Pathogenic |
| ***JAK2*** | c.1849G>T | p.(Val617Phe) | 20,3 | Missense | Pathogenic |
| ***JAK2*** | c.1849G>T | p.(Val617Phe) | 19,6 | Missense | Pathogenic |
| ***JAK2*** | c.1849G>T | p.(Val617Phe) | 58,1 | Missense | Pathogenic |
| ***JAK2*** | c.1849G>T | p.(Val617Phe) | 50,2 | Missense | Pathogenic |
| ***JAK2*** | c.1849G>T | p.(Val617Phe) | 31,9 | Missense | Pathogenic |
| ***JAK2*** | c.1849G>T | p.(Val617Phe) | 16,8 | Missense | Pathogenic |
| ***JAK2*** | c.1849G>T | p.(Val617Phe) | 4,7 | Missense | Pathogenic |
| ***JAK2*** | c.1849G>T | p.(Val617Phe) | 60,3 | Missense | Pathogenic |
| ***JAK2*** | c.1849G>T | p.(Val617Phe) | 2,0 | Missense | Pathogenic |
| ***JAK2*** | c.1849G>T | p.(Val617Phe) | 84,7 | Missense | Pathogenic |
| ***JAK2*** | c.1849G>T | p.(Val617Phe) | 23,6 | Missense | Pathogenic |
| ***JAK2*** | c.1849G>T | p.(Val617Phe) | 18,1 | Missense | Pathogenic |
| ***JAK2*** | c.1849G>T | p.(Val617Phe) | 54,1 | Missense | Pathogenic |
| ***JAK2*** | c.1849G>T | p.(Val617Phe) | 23,1 | Missense | Pathogenic |
| ***JAK2*** | c.1849G>T | p.(Val617Phe) | 22,4 | Missense | Pathogenic |
| ***JAK2*** | c.1849G>T | p.(Val617Phe) | 39,5 | Missense | Pathogenic |
| ***JAK2*** | c.1849G>T | p.(Val617Phe) | 15,4 | Missense | Pathogenic |
| ***JAK2*** | c.1849G>T | p.(Val617Phe) | 50,3 | Missense | Pathogenic |
| ***JAK2*** | c.1849G>T | p.(Val617Phe) | 67,4 | Missense | Pathogenic |
| ***JAK2*** | c.1849G>T | p.(Val617Phe) | 19,0 | Missense | Pathogenic |
| ***JAK2*** | c.1849G>T | p.(Val617Phe) | 85,9 | Missense | Pathogenic |
| ***JAK2*** | c.1849G>T | p.(Val617Phe) | 92,0 | Missense | Pathogenic |
| ***JAK2*** | c.1849G>T | p.(Val617Phe) | 83,7 | Missense | Pathogenic |
| ***JAK2*** | c.1849G>T | p.(Val617Phe) | 75,3 | Missense | Pathogenic |
| ***JAK2*** | c.1849G>T | p.(Val617Phe) | 40,1 | Missense | Pathogenic |
| ***JAK2*** | c.1849G>T | p.(Val617Phe) | 92,9 | Missense | Pathogenic |
| ***JAK2*** | c.1849G>T | p.(Val617Phe) | 51,4 | Missense | Pathogenic |
| ***JAK2*** | c.1849G>T | p.(Val617Phe) | 65,6 | Missense | Pathogenic |
| ***JAK2*** | c.1849G>T | p.(Val617Phe) | 66,3 | Missense | Pathogenic |
| ***JAK2*** | c.1849G>T | p.(Val617Phe) | 46,6 | Missense | Pathogenic |
| ***JAK2*** | c.1849G>T | p.(Val617Phe) | 38,5 | Missense | Pathogenic |
| ***JAK2*** | c.1849G>T | p.(Val617Phe) | 10,8 | Missense | Pathogenic |
| ***JAK2*** | c.1849G>T | p.(Val617Phe) | 92,5 | Missense | Pathogenic |
| ***JAK2*** | c.1849G>T | p.(Val617Phe) | 22,7 | Missense | Pathogenic |
| ***JAK2*** | c.1849G>T | p.(Val617Phe) | 5,2 | Missense | Pathogenic |
| ***JAK2*** | c.1849G>T | p.(Val617Phe) | 7,1 | Missense | Pathogenic |
| ***JAK2*** | c.1849G>T | p.(Val617Phe) | 15,0 | Missense | Pathogenic |
| ***JAK2*** | c.1849G>T | p.(Val617Phe) | 40,5 | Missense | Pathogenic |
| ***JAK2*** | c.1849G>T | p.(Val617Phe) | 32,3 | Missense | Pathogenic |
| ***JAK2*** | c.1849G>T | p.(Val617Phe) | 6,8 | Missense | Pathogenic |
| ***JAK2*** | c.1849G>T | p.(Val617Phe) | 73,6 | Missense | Pathogenic |
| ***JAK2*** | c.1849G>T | p.(Val617Phe) | 20,9 | Missense | Pathogenic |
| ***JAK2*** | c.1849G>T | p.(Val617Phe) | 31,2 | Missense | Pathogenic |
| ***JAK2*** | c.1849G>T | p.(Val617Phe) | 93,2 | Missense | Pathogenic |
| ***JAK2*** | c.1849G>T | p.(Val617Phe) | 23,7 | Missense | Pathogenic |
| ***JAK2*** | c.1849G>T | p.(Val617Phe) | 78,6 | Missense | Pathogenic |
| ***JAK2*** | c.1849G>T | p.(Val617Phe) | 35,2 | Missense | Pathogenic |
| ***JAK2*** | c.1849G>T | p.(Val617Phe) | 35,2 | Missense | Pathogenic |
| ***JAK2*** | c.1849G>T | p.(Val617Phe) | 16,0 | Missense | Pathogenic |
| ***JAK2*** | c.1849G>T | p.(Val617Phe) | 37,3 | Missense | Pathogenic |
| ***JAK2*** | c.1849G>T | p.(Val617Phe) | 15,2 | MIssense | Pathogenic |
| ***JAK2*** | c.1849G>T | p.(Val617Phe) | 56,4 | Missense | Pathogenic |
| ***JAK2*** | c.1849G>T | p.(Val617Phe) | 70,5 | Missense | Pathogenic |
| ***JAK2*** | c.1849G>T | p.(Val617Phe) | 2,3 | Missense | Pathogenic |
| ***JAK2*** | c.1849G>T | p.(Val617Phe) | 40,8 | Missense | Pathogenic |
| ***JAK2*** | c.1849G>T | p.(Val617Phe) | 20,8 | Missense | Pathogenic |
| ***JAK2*** | c.1849G>T | p.(Val617Phe) | 53,3 | Missense | Pathogenic |
| ***JAK2*** | c.1849G>T | p.(Val617Phe) | 22,5 | Missense | Pathogenic |
| ***JAK2*** | c.1849G>T | p.(Val617Phe) | 29,9 | Missense | Pathogenic |
| ***JAK2*** | c.1849G>T | p.(Val617Phe) | 42,8 | Missense | Pathogenic |
| ***JAK2*** | exón 12 | p.His538_Lys539delinsGln | 2,1 | Frameshift | Pathogenic |
| ***KRAS*** | c.108A>G | p.(Ile36Met) | 16,7 | Missense | Likely pathogenic |
| ***MPL*** | c.1771T>G | p.(Tyr591Asp) | 8,7 | Missense | Likely pathogenic |
| ***MPL*** | c.610T>C | p.(Ser204Pro) | 39,6 | Missense | Likely pathogenic |
| ***SF3B1*** | c.1997A>G | p.(Lys666Arg) | 47,8 | Missense | Pathogenic |
| ***SF3B1*** | c.1998G>C | p.(Lys666Arg) | 39,8 | Missense | Pathogenic |
| ***SF3B1*** | c.1998G>T | p.(Lys666Asn) | 41,9 | Missense | Pathogenic |
| ***SF3B1*** | c.2098A>G | p.(Lys700Glu) | 9,8 | Missense | Pathogenic |
| ***SF3B1*** | c.1998G>T | p.(Lys666Asn) | 40,1 | Missense | Pathogenic |
| ***SRSF2*** | c.284C>T | p.(Pro95Leu) | 49,8 | Missense | Pathogenic |
| ***SRSF2*** | c.284C>G | p.(Pro95Arg) | 43,8 | Missense | Pathogenic |
| ***SRSF2*** | c.284C>T | p.(Pro95Leu) | 46,0 | Missense | Likely pathogenic |
| ***TET2*** | c.1337del | p.(Leu446*) | 3,7 | Nonsense | Pathogenic |
| ***TET2*** | c.3812dupG | p.(Cys1271Trpfs*29) | 4,6 | Frameshift | Pathogenic |
| ***TET2*** | c.3812dupG | p.(Cys1271Trpfs*29) | 38,4 | Frameshift | Pathogenic |
| ***TET2*** | c.4879C>T | p.(Gln1627*) | 7,6 | Nonsense | Pathogenic |
| ***TET2*** | c.4062_4063del | p.(Arg1354Serfs*46) | 28,1 | Frameshift | Pathogenic |
| ***TET2*** | c.3903del | p.(Arg1302Glufs*61) | 28,9 | Frameshift | Pathogenic |
| ***TET2*** | c.1531del | p.(His511Thrfs*22) | 6,2 | Frameshift | Pathogenic |
| ***TET2*** | c.385C>T | p.(Gln129*) | 15,5 | Nonsense | Likely pathogenic |
| ***TET2*** | c.4678del | p.(Tyr1560Ilefs*11) | 39,5 | Frameshift | Likely pathogenic |
| ***TET2*** | c.3637del | p.(Val1213Cysfs*13) | 3,1 | Frameshift | Likely pathogenic |
| ***TET2*** | c.4590_4604del | p.(Leu1531_Pro1535del) | 47,0 | In frame | Likely pathogenic |
| ***TET2*** | c.3686T>G | p.(Leu1229Arg) | 44,0 | Missense | Likely pathogenic |
| ***TET2*** | c.1526C>G | p.(Ser509*) | 5,3 | Nonsense | Likely pathogenic |
| ***TET2*** | c.4249G>T | p.(Val1417Phe) | 77,2 | Missense | Likely pathogenic |
| ***TET2*** | c.2708del | p.(Asn903Thrfs*18) | 20,2 | Frameshift | Likely pathogenic |
| ***TET2*** | c.195_201del | p.(Cys65Trpfs*8) | 45,7 | Frameshift | Likely pathogenic |
| ***TET2*** | c.3640C>T | p.(Arg1214Trp) | 49,4 | Missense | Likely pathogenic |
| ***TET2*** | c.2896C>T | p.(Gln966*) | 2,4 | Nonsense | Likely pathogenic |
| ***TET2*** | c.822del | p.(Asn275Ilefs*18) | 2,0 | Frameshift | Likely pathogenic |
| ***TET2*** | c.4523_4524del | p.(Ala1508Glufs*69) | 3,6 | Frameshift | Likely pathogenic |
| ***TET2*** | c.2650C>T | p.(Gln884*) | 3,1 | Nonsense | Likely pathogenic |
| ***TET2*** | c.4138C>T | p.(His1380Tyr) | 2,5 | Missense | Likely pathogenic |
| ***TET2*** | c.2314_2315del | p.(Glu772Argfs*8) | 2,7 | Frameshift | Likely pathogenic |
| ***TET2*** | c.3764dupA | p.(Tyr1255*) | 4,1 | Nonsense | Likely pathogenic |
| ***TET2*** | c.3466_3481del | p.(Asn1156Glufs*65) | 11,0 | Frameshift | Likely pathogenic |
| ***TET2*** | c.2332C>T | p.(Gln778*) | 17,5 | Nonsense | Likely pathogenic |
| ***TET2*** | c.671_678del | p.(Glu224Glyfs*27) | 6,3 | Frameshift | Likely pathogenic |
| ***TET2*** | c.5618T>C | p.(Ile1873Thr) | 46,3 | Missense | Likely pathogenic |
| ***TET2*** | c.3640C>T | p.(Arg1214Trp) | 14,6 | Missense | Likely pathogenic |
| ***TET2*** | c.3965T>G | p.(Leu1322Arg) | 34,5 | Missense | Likely pathogenic |
| ***TET2*** | c.1897dupA | p.(Met633Asnfs*5) | 7,7 | Frameshift | Likely pathogenic |
| ***TET2*** | c.4935T>G | p.(Tyr1645*) | 49,3 | Nonsense | Likely pathogenic |
| ***TET2*** | c.154del | p.(Thr52Profs*15) | 9,8 | Frameshift | Likely pathogenic |
| ***TET2*** | c.2474del | p.(Ser825*) | 23,0 | Nonsense | Likely pathogenic |
| ***TET2*** | c.2686del | p.(Leu896Tyrfs*25) | 3,8 | Frameshift | Likely pathogenic |
| ***TET2*** | c.3444:3445del | p.(Thr1149Profs*7) | 37,1 | Frameshift | Likely pathogenic |
| ***TET2*** | c.4990C>T | p.(Gln1664*) | 3,0 | Nonsense | Likely pathogenic |
| ***TET2*** | c.1544del | p.(Asn515Thrfs*18) | 2,7 | Frameshift | Likely pathogenic |
| ***TET2*** | c.3767del | p.(Gly1256Alafs*10) | 3,2 | Frameshift | Likely pathogenic |
| ***TET2*** | c.5618T>C | p.(Ile1873Thr) | 45,3 | Missense | Likely pathogenic |
| ***TET2*** | c.2674C>T | p.(Gln892*) | 10,1 | Nonsense | Likely pathogenic |
| ***TET2*** | c.4097G>A | p.(Arg1366His) | 41,0 | Missense | Likely pathogenic |
| ***TET2*** | c.2392G>T | p.(Glu798*) | 18,5 | Nonsense | Likely pathogenic |
| ***TET2*** | c.2643del | p.(Arg881Serfs*40) | 7,4 | Frameshift | Likely pathogenic |
| ***TP53*** | c.763A>T | p.(Ile255Phe) | 2,8 | Missense | Pathogenic |
| ***TP53*** | c.818G>A | p.(Arg273His) | 9,0 | Missense | Pathogenic |
| ***TP53*** | c.743G>A | p.(Arg248Gln) | 85,1 | Missense | Pathogenic |
| ***TP53*** | c.743G>A | p.(Arg248Gln) | 42,4 | Missense | Pathogenic |
| ***TP53*** | c.770T>A | p.(Leu257Gln) | 19,5 | Missense | Likely pathogenic |
| ***TP53*** | c.542G>A | p.(Arg181His) | 2,5 | Missense | Likely pathogenic |
| ***TP53*** | c.917G>A | p.(Arg306Gln) | 46,3 | MIssense | Likely pathogenic |
| ***TP53*** | c.658T>C | p.(Tyr220His) | 6,8 | Missense | Likely pathogenic |
| ***TP53*** | c.783-1G>T |  | 26,5 | Splicing | Likely pathogenic |
| ***TP53*** | c.614A>G | p.(Tyr205Cys) | 1,5 | Missense | Likely pathogenic |
| ***TP53*** | c.644G>A | p.(Ser215Asn) | 3,5 | Missense | Likely pathogenic |
| ***U2AF1*** | c.470A>G | p.(Gln157Arg) | 36,1 | Missense | Pathogenic |
| ***U2AF1*** | c.101C>T | p.(Ser34Phe) | 38,3 | Missense | Pathogenic |
| ***U2AF1*** | c.470A>G | p.(Gln157Arg) | 39,1 | Missense | Pathogenic |

**Supplemental Table S3. Proportion of patients receiving each cytoreductive therapy in each group**

|  | **MPN without SVT,**  ***n*=165** | **MPN presenting with SVT or previous SVT¶,**  ***n*=69** | **MPN with evolutive SVT, *n*=21** | **Total, *n*=255** | ***p* value†** |
| --- | --- | --- | --- | --- | --- |
| **Hydroxyurea** | 69.1% | 74.6% | 90.5% | 72.5% | 0.19 |
| **Anagrelide** | 8.5% | 14.3% | 15.8% | 13.7% | 0.56 |
| **Interferon** | 12.7% | 10.2% | 9.5% | 11.8% | 0.94 |
| **JAK inhibitors** | 7.3% | 10.2% | 23.8% | 9.4% | 0.11 |
| **P32** | 4.8% | 1.7% | 4.8% | 3.9% | 0.66 |
| **Busulfan** | 3% | 0% | 0% | 2% | 0.43 |

Abbreviations: MPN, myeloproliferative neoplasm; SVT, splanchnic vein thrombosis.

†*p* value, as a result of comparison of the three groups
